# Electrophysiological correlates of motor sequence learning

**DOI:** 10.1186/1471-2202-15-102

**Published:** 2014-08-28

**Authors:** Christelle Beaulieu, Marie-Ève Bourassa, Benoit Brisson, Pierre Jolicoeur, Louis De Beaumont

**Affiliations:** Université du Québec à Trois Rivières, C.P. 5000, Trois Rivières, Québec G9A 5H7 Canada; Université de Montréal, C.P. 6205, Succursale Centre-ville, Montréal, Québec H3C 3T5 Canada; Montreal Sacred Heart Hospital Research Centre, 5400, Boulevard Gouin Ouest, Montréal, Québec H4J1C5 Canada

**Keywords:** Event-related potentials (ERPs), Error-related Negativity (ERN), Cognitive control, Motor learning, Serial reaction time task

## Abstract

**Background:**

The *Error-related negativity* (ERN) is a component of the event-related brain potentials elicited by error commission. The ERN is thought to reflect cognitive control processes aiming to improve performance. As previous studies showed a modulation of the ERN amplitude throughout the execution of a learning task, this study aims to follow the ERN amplitude changes from early to late learning blocks in relation with concomitant motor sequence learning using a serial reaction time (SRT) task. Twenty-two healthy participants completed a SRT task during which continuous EEG activity was recorded. The SRT task consists of series of stimulus-response pairs and involves motor learning of a repeating sequence. Learning was computed as the difference in mean response time between the last sequence block and the last random blocks that immediately follows it (sequence-specific learning). Event-related potentials were analysed to measure ERN amplitude elicited by error commission.

**Results:**

Mean ERN amplitude difference between the first four learning blocks and the last four learning blocks of the SRT task correlated significantly with motor sequence learning as well as with overall response time improvement, such that those participants whose ERN amplitude most increased through learning blocks were also those who exhibited most SRT task improvements. In contrast, neither sequence-specific learning nor overall response time improvement across learning blocks were found to be related to averaged ERN amplitude from all learning blocks.

**Conclusion:**

Findings from the present study suggest that the ERN amplitude changes from early to late learning blocks occurring over the course of the SRT task, as opposed to the averaged ERN amplitude from all learning blocks, is more closely associated with learning of a motor sequence. These findings propose an improved electrophysiological marker to index change in cognitive control efficiency during motor sequence learning.

## Background

Cognitive control refers to the ability to adopt goal-directed behaviors by regulating cognitive processes. It is a system linked to higher-level cognitive functions such as attention, planning, inhibition, and cognitive flexibility [1]. It contributes to the detection of conflict situations and erroneous responses in order to allocate cognitive resources to the implementation of adaptive strategies [2, 3].

A neurophysiological correlate of such cognitive control system was first observed by Hohnsbein, Falkenstein, Hoormann and Blanke [4] in what they called the *Error Negativity* (Ne), which is now also commonly referred to as the *Error-related Negativity* [ERN; [5]]. The ERN is a component of the event-related brain potential (ERP) elicited by error commission. It is generally known as an index of the evaluative processes of cognitive control involved in one’s own performance monitoring [2, 4, 5]. This component is usually maximal at central and frontocentral electrode positions (Cz and FCz in the standard 10–10 system of electroencephalogram (EEG) electrode placement) and appears as a negative deflection that peaks within 0–100 ms following an erroneous overt response. Frequency-domain studies suggest that anterior theta (4–8 Hz) EEG activity could underlie the generation of this ERP waveform [6–9].

The mismatch hypothesis was the first of three influential theories attempting to account for the nature and role of the ERN in relation to cognitive control [4, 5]. The latter theory suggests that a comparator system continuously evaluates the similarities between the neural representations of the initiated response and the correct response. When the subject makes an erroneous response, a discrepancy between these neural representations is perceived, which generates the ERN. In the reinforcement learning theory [10], performance monitoring relies on the evaluation of the action outcome, that is either better or worse than expected. The ERN would thus occur when expectations are violated. The authors argue that the processes underlying this component contribute to the initiation of adaptive strategies that enables the correction or reduction of the number of errors. Alternatively, the conflict monitoring hypothesis [2] proposes that the error-related waveform is rather elicited by a simultaneous activation of more than one incompatible responses in a conflict situation, as it is the case in experimental paradigms such as the Stroop or the Eriksen Flanker paradigm [11, 12], that manipulate the congruency between stimulus and response. The ERN would thus reflect a monitoring process that signals the need for an increased control on actions in order to maintain adequate performance levels.

Studies focusing on the neural generator of the error-related activity point to the anterior cingulate cortex (ACC) and the supplementary motor area (SMA) was the probable neuronal generators of the ERN [10, 13]. Both the reinforcement learning and the conflict monitoring theories argue for a predominant role of the ACC in the generation of the ERN. The former proposes that the mesencephalic dopamine system contributes to learning via phasic transmissions of dopamine to the prefrontal cortex, resulting in a reinforcement signal. However, an action outcome worse than expected reduces this reinforcement signal, leading to ACC-regulated disinhibition of motor neurons, which generate the error-related activity recording on the scalp [10]. For the latter, the ERN occurs when a conflict in response activation is detected and reflects a signal transmitted by the ACC to the prefrontal cortex for higher-level cognitive functions [2, 14].

In the last two decades, research on factors modulating the ERN response has received a growing interest. The motivational significance of an error has been suggested as a powerful mediator of the ERN response [5, 11]. In parallel, studies showed that the amplitude of the ERN predicts post-error slowing [15] and that both amplitude and latency of the ERN are associated with a subsequent correction of a committed error [16–18]. Taken together, these results suggest that cognitive operations reflected by the ERN support the initiation of top-down processes aiming to improve performance.

In neurologically intact participants, studies showed that psychological symptoms such as anxiety and negative affect, personality traits such as impulsiveness, and even variables such as «level of satisfaction toward life», predict individual differences in cognitive control efficiency and ERN amplitude [19–22]. The ERN waveform component was also measured in clinical populations suspected to present with cognitive control alterations. Notably, obsessive-compulsive disorder (OCD) patients exhibit significantly increased ERN amplitude [23], whereas similar experimental paradigms applied to schizophrenia as well as traumatic brain injury (TBI) rather elicited ERN waveform components of smaller amplitude [24, 25]. This opposite pattern of ERN amplitude reflects the anticipated performance monitoring excess in OCD patients as opposed to performance monitoring deficiency in TBI and schizophrenic patients. Limited clinical significance, however, has been granted to ERN amplitude changes because potential associations with clinically validated symptom measures or behavioral performance have yet to be demonstrated. Knowing that the ERN is modulated by a variety of factors (extraneous to clinical symptomatology) that may continuously evolve during task performance, a close monitoring of the change of the ERN component throughout task performance could help improve its association with behavioral performance scores, thereby improving its clinical utility.

The serial reaction time task (SRT) has been used to generate clearly discernible ERN waveform components to erroneous response. A SRT task consists of a series of stimulus-response pairs and often involves implicit (i.e., participants are not informed about the existence of the sequence) motor learning of a repeating motor sequence arranged in blocks inserted in otherwise random sequence blocks. In this task, learning is characterized by a progressive reduction in response time (RT) as participants complete a series of learning blocks relative to random sequence blocks. The rapid execution and short response-stimulus intervals in this paradigm increase the number of effective observations and can promote the generation of errors when a presented sequence deviates from a learned sequence. A study by Holroyd and Coles [10] looked at the modulation of the ERN relative to dynamic learning in a probabilistic learning task. Results from correlational analyses showed that the amplitude of the ERN tended to increase with the number of learning blocks. In a trial-and-error motor learning task, the ERN amplitude following a feedback (commonly referred to as the feedback-related negativity; FRN) has been shown to predict motor learning efficiency [26]. In this study, an enhanced FRN was found for errors that were subsequently adjusted compared to errors that were repeated. These results suggest that a motor sequence learning task including numerous learning blocks, such as the SRT task proposed by Perez, Wise, Willingham and Cohen [27], may allow one to follow the changes of the ERN response in relation with concomitant behavioral performance modifications.

Perhaps the most advantageous feature of the SRT task to investigate ERN brain response with concomitant learning is the ability to discriminate sequence-specific learning from general RT improvements. Sequence-specific learning is reflected in RT difference between the last sequence-learning block and the random block that immediately follows it, such that adequate control for habituation effects with the task procedure, overall key press facilitation, motivational effects, as well as vigilance or fatigue is provided. Such sequence-specific learning measure has proved to be strongly associated with neurophysiological measures of primary motor cortex (M1) synaptic plasticity [28], a relation that could not be established with traditional motor sequence learning effects (i.e., computing RT differences between the first learning block and the last learning block). Along those lines, no study has looked at the association between sequence-specific learning and ERN amplitude changes from early learning blocks to late learning blocks. Here, we explore the possibility that participants with greater ERN amplitude in late learning blocks relative to early learning blocks will be those who exhibit most sequence-specific motor learning.

## Results

### Behavioral results

**Figure 1:**
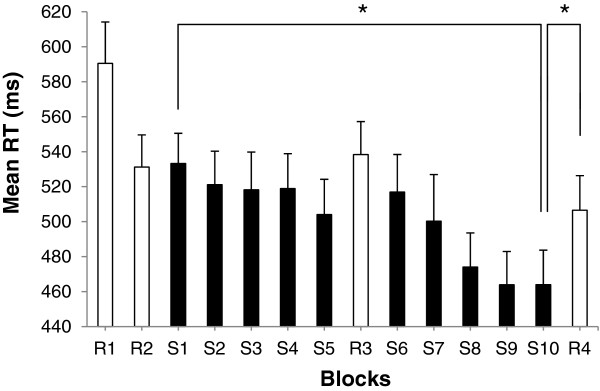
**Mean response time (RT) in random (R**_**1-4**_ **) and learning block (S**_**1-10**_ **) during the SRT task.** The abscissa shows block type in temporal order, and the ordinate shows mean RT. Error bars display standard error values. Asterisk (*) corresponds to *p* < .001.

Whole-group averaged RT for each block of the SRT task is presented in Figure 1. As expected, significant RT improvements were found when comparing the first learning block with the last learning block (S1-S10) (*t* (21) = 5.998; *p* < .001), as well as when computing sequence-specific learning (R4-S10) (*t* (21) = 4.651; *p* < .001). Mean RT recorded for S_1-4_ significantly differed from mean RT for S_7-10_ (*t* (21) = 6.033; *p* < .001). Accuracy did not vary, neither across learning blocks [S1: 95.5% ± 3.3; S10: 94.6% ± 3.6; (*t* (21) = 1.447; *p* > .05)], nor across sets of learning blocks [S_1-4_: 94.8% ± 2.7; S_7-10_: 94.6% ± 2.3; (*t* (21) = .703; *p* > .05)].

### Electrophysiological results

**Figure 2:**
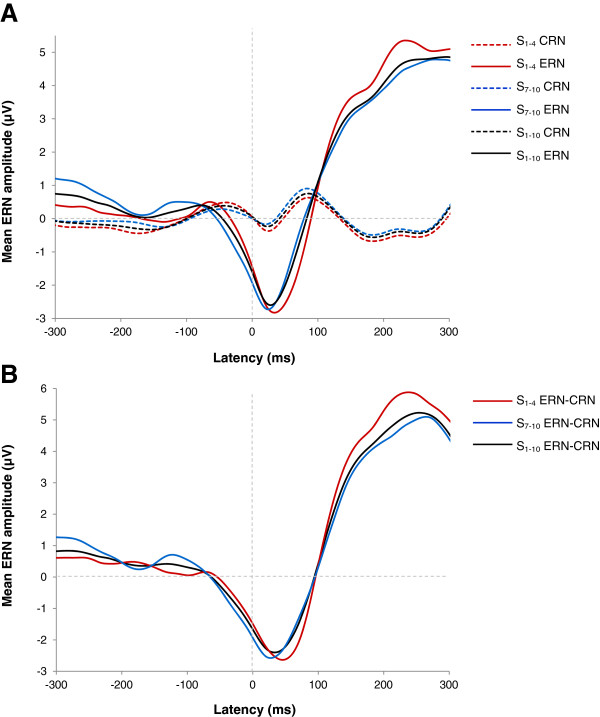
**Grand averages of error-related negativity (ERN) and correct-related negativity (CRN) waveforms timelocked to the subject’s response. A**: CRN and ERN estimated at Cz and FCz for the first four learning blocks (S_1-4_), the last four learning blocks (S_7-10_) and all learning blocks (S_1-10_). **B**: Subtracted ERN (ERN-CRN) estimated at Cz and FCz for the first four learning blocks (S_1-4_), the last four learning blocks (S_7-10_) and all learning blocks (S_1-10_).

On average, subtracted ERN components (ERN - CRN) were based on 301.1 correct trials (SD = 78.23) and 17.1 error trials (SD = 9.8) per subject for the first set of learning blocks (S_1-4_) and on 321.7 correct trials (SD = 77.29) and 18.6 error trials (SD = 9.01) per participant for the last set of learning blocks (S_7-10_). The number of committed errors for S_1-4_ varied between 6 and 51, while that for S_7-10_ varied between 6 and 43. Figure 2 illustrates grand averages of CRN, ERN and subtracted ERN waveforms (ERN - CRN) estimated from pooled electrodes Cz and FCz for S_1-4_, S_7-10_, and S_1-10_. The latency of the ERN was found to be significantly shorter for S_7-10_ than for S_1-4_ (*t* (21) = 3.485; *p* = .002). In contrast, ERN amplitude recordings from S_1-4_ did not significantly differ from S_7-10_ recordings (*t* (21) = -1.706; *p* > .05).

**Figure 3:**
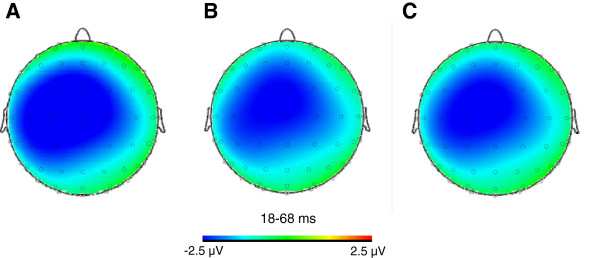
**Scalp topographies of the subtracted error-related negativity (ERN; ERN - CRN) between 18 and 68 ms. A**: For the first four learning block (S_1-4_). **B**: For the last four learning blocks (S_7-10_). **C**: For all learning blocks (S_1-10_).

Figure 3 shows scalp topographies of the mean electric brain activity during the response-locked 18-68 ms time window across learning block conditions (i.e., S_1-4_, S_7-10_, S_1-10_). The selected time window corresponds to the time span during which the grand averaged S_1-10_ ERN waveform component was of maximal amplitude.

### Correlational analyses

**Figure 4:**
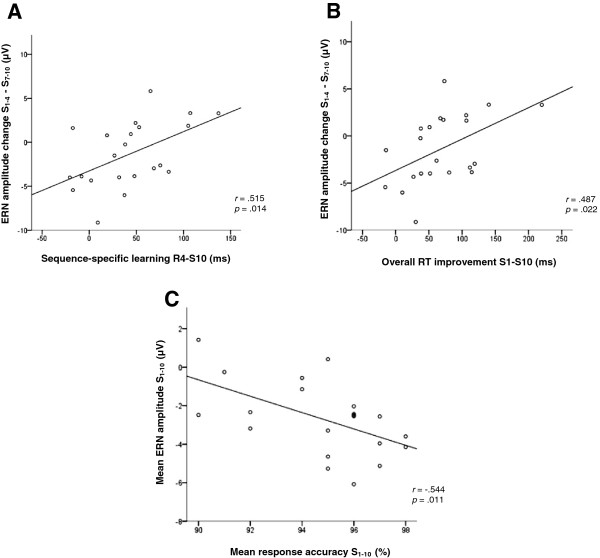
**Correlational results. A**: Correlation between S_1-4_ - S_7-10_ ERN amplitude difference (μV) recorded at Cz and sequence-specific learning (R4-S10) (ms). **B**: Correlation between S_1-4_ - S_7-10_ ERN amplitude difference (μV) recorded at Cz and overall response time (RT) improvement (S1-S10). **C**: Correlation between mean S_1-10_ ERN amplitude (μV) recorded at Cz and mean S_1-10_ response accuracy (%).

For each subject, we first computed sequence-specific learning (R4-S10) and then conducted Pearson correlations with ERN amplitude difference between the first four learning blocks and the last four learning blocks (S_1-4_ - S_7-10_). The evolution of the ERN across learning blocks was found to significantly correlate with sequence-specific learning (*r* = .515; *p* = .014) (Figure 4A). A similar correlational analysis looking at overall RT improvement (S1-S10) in relation with the evolution of the ERN was also significant (*r* = .487; *p* = .022) (Figure 4B), such that those participants whose ERN amplitude most increased through learning blocks were also those who exhibited most SRT task improvements. In contrast, similar correlations between S_1-4_ - S_7-10_ ERN latency difference and **1-** Sequence-specific learning (R4-S10) (*r* = -.122; *p* > .05); **2-** Overall RT improvement (S1-S10) (*r* = .128; *p* > .05); and **3-** S_1-4_ - S_7-10_ ERN amplitude difference (*r* = -.063; *p* > .05) were not significant. Consistent with the literature, a negative correlation was found between the S_1-10_ mean ERN amplitude and the S_1-10_ mean response accuracy (*r* = -.544; *p* = .011) *(*Figure 4C), such that larger ERN size is associated with better response accuracy. However, similar relations between S_1-10_ mean ERN amplitude could neither be found with sequence-specific learning (R4-S10) (*r* = .192; *p* > .05) nor overall RT improvement (S1-S10) (*r* = .311; *p* > .05).

**Figure 5:**
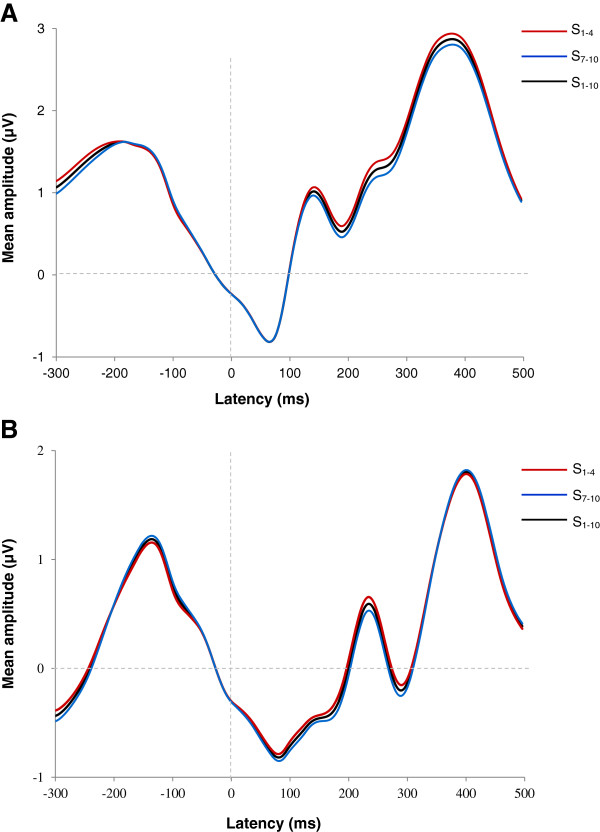
**Grand averages of P1, N1 and P3 waveforms timelocked to the stimuli. A**: P1 and N1 waveforms estimated at PO7 and PO8 for the first four learning blocks (S_1-4_), the last four learning blocks (S_7-10_) and all learning blocks (S_1-10_). **B**: P3 waveforms estimated at Pz for the first four learning blocks (S_1-4_), the last four learning blocks (S_7-10_) and all learning blocks (S_1-10_).

Post-hoc analyses were conducted to assess potential associations between sequence-specific learning (R4-S10), overall RT improvement (S1-S10) and dynamic amplitude changes in ERP components implicated in early and late stages of perceptual processing. Specifically, P1 and N1 are visually evoked potentials reflecting attentional processes, while the P3 waveform component is referred to as an index of working memory updating and attentional resources allocation [29, 30]. P1, N1, and P3 mean amplitudes changes (S_1-4_ - S_7-10_) were computed using a predefined stimulus-locked time window of 115–155 ms, 165–205 ms and 365–405 ms, respectively. The N1 and P1 amplitudes were both estimated at pooled electrodes PO7 and PO8, whereas the P3 amplitude was estimated at electrode Pz (Figure 5). These electrodes were chosen as they recorded maximal brain activity for each of the targeted ERP components. ERP component changes with task progression were unrelated to sequence-specific learning (R4-S10) (P1 (*r* = -.121; *p* > .05), N1 (*r* = .05; *p* > .05) or P3 (*r* = .006; *p* > .05)). Similarly, overall RT improvement (S1-S10) did not correlate with P1 changes (*r* = .149; *p* > .05), N1 changes (*r* = .109; *p* > .05) or P3 changes (*r* = -.068; *p* > .05).

## Discussion

The main finding of this study is the demonstration that an increase of ERN amplitude from early to late learning blocks is strongly associated with RT reduction in a SRT task designed to investigate implicit motor sequence learning. Interestingly, this association with the ERN component shows some specificity given that other waveform components implicated in different stages of information processing (i.e. N1, P1, and P3) were found to be unrelated with RT improvements. The association between learning of a motor sequence and the index of ERN amplitude change revealed herein significantly contrasts with conventional, grand averaged ERN amplitude or latency approaches, which consistently revealed no association with SRT task response time [15–18, 31]. By tracking the ERN amplitude changes from early to late learning blocks (early − late ERN changes) within a SRT task procedure, findings from the present study propose an improved electrophysiological marker to index changes in cognitive control efficiency during motor sequence learning.

To our knowledge, few studies have specifically looked at the modulation of the ERN component with concomitant behavioral performance measures during a motor learning task. The demonstration that greater RT improvements at the SRT task are found in participants whose ERN amplitude increases with task progression is consistent with previous correlational evidence derived from probabilistic/perceptual studies [10, 32]. The increase in the amplitude of the ERN is thought to reflect the continuous acquisition of the ability to evaluate response errors internally within the course of a speeded task [32].

Prior studies suggest an association between mean ERN amplitude (or FRN amplitude) and learning strategies efficiency [15, 18, 26]. In the current study, mean ERN amplitude derived from all learning blocks (S_1-10_) of the SRT task was found to be related to mean response accuracy, consistent with the notion that error monitoring might shape future behaviors intended to enhance performance [20, 33, 34]. In contrast, mean S_1-10_ ERN amplitude from the present study did not correlate with RT improvements on either overall learning (S1-S10) or sequence-specific learning (R4-S10) conditions. Sequence-specific RT improvements were rather found to associate exclusively with incremental ERN amplitude increase occurring with task progression. Taken together, these findings suggest that the early − late ERN changes taking place over the course of the SRT task, as opposed to the mean ERN amplitude from all learning blocks, is more closely associated with learning of a motor sequence.

Based on the reinforcement learning theory [10], the ERN is elicited by an internal or an external feedback and represents the primary signal that an action outcome is worse than expected. This signal is then used to train the response production system to optimize performance. In contrast with feedback-dependent trial-and-error learning task, in which participants discover a stimulus-response mapping [26, 32, 35], participants in the present study learned a repeating motor sequence based on a stimulus-response mapping presented prior to the beginning of the experiment. Participants could therefore rely on their own internal representation of the correct response to assess their performance. Accordingly, the association between ERN amplitude increase and sequence-specific RT improvements may reflect that an error committed at the end of the task could be perceived as two errors, that is, an error in the visuomotor stimulus-response mapping and an error in the motor sequence to be executed. Alternatively, according to the mismatch hypothesis [5], the ERN is elicited when a comparator system perceives a discrepancy between the neural representation of the correct response and the initiated response. In the SRT task, the numerous repetitions of the motor sequence to execute generate learning which may allow a stronger internalization of the correct response pattern. Therefore, for those participants who exhibit most learning at the SRT task, an error made at the end of the task may lead to a greater discrepancy between the neural representations of the correct response and the erroneous response and, consequently, to a larger ERN. In parallel, the significantly shortened ERN latency found toward the end of the SRT task can be explained by a participant foreseeing the next motor response to be executed, thus creating a time lag between response determination process and the overt motor response. Importantly, prior studies suggest that response choice rather than the motor response itself elicits the ERN [36, 37]. It therefore stands to reason that anticipation of the next finger press was greater after having completed six blocks of a repeating motor sequence, thus precipitating the generation of the ERN waveform component.

Functional connectivity variability in a healthy population could partially account for the relation found between the early − late ERN changes and learning of a motor sequence. Indeed, recent studies evidenced a distributed network for error monitoring and cognitive control that capitalizes on coordinated theta oscillations for local and long-range functional connectivity [6, 38]. In parallel, synchronized theta oscillations are associated with motor performance improvement and learning-dependent synaptic plasticity mechanisms [39–41]. A recent study using a speeded flanker task found significant theta synchrony between medial frontal electrode sites — i.e. where maximal ERN activity is recorded — and left central electrode sites, respectively associated with performance monitoring and motor execution [42]. These authors suggested that this synchrony might reflect control functions during conflict resolution between competing motor activations. Along those lines, participants who showed greatest task improvements at a time estimation task exhibited larger theta connectivity from left central to mid-frontal electrode sites following feedback [43]. These findings point to the central role of efficient motor to mid-frontal connectivity in cognitive control. In keeping with these findings, scalp topographies from the present study illustrating averaged ERN recordings from 64 electrodes scattered over the scalp show a lateralization of cerebral activation toward the left hemisphere. This hemispheric lateralization is spatially concordant with the left M1 contralateral to the hand solicited for SRT task execution. It therefore stands to reason that the relationship between sequence-specific learning and ERN amplitude could be the result of functional connectivity between involved cortical areas occurring with task execution. Previous studies have suggested that M1 synaptic plasticity mechanisms are at least partially implicated in both learning of a repeating motor sequence and concomitant functional connectivity changes [44]. Further connectivity analyses, however, are needed to validate this hypothesis.

## Conclusion

The present study aimed to monitor early − late ERN changes in relation with motor sequence learning occurring within a SRT task. Our findings suggest that the increased ERN amplitude from early to late learning blocks of a repeating motor sequence is significantly associated with sequence-specific RT improvements. This electrophysiological marker of dynamic cognitive control changes offers clear advantages over typical, grand average ERN amplitude calculations as it appears to more closely associate with motor sequence learning. Future applications of this ERN amplitude index should be explored in various tasks involving learning, particularly among clinical populations experiencing cognitive control deficiencies. To this end, future studies should collect test-retest reliability measures of the ERN amplitude change index proposed herein in order to assess its applicability to clinical settings.

## Methods

### Participants

Twenty-five participants completed the experiment. Data from three participants were excluded from further analysis, two for an insufficient number of committed errors (less than 6 errors) and one based on pre-determined data contamination criteria (refer to the section on Event-related potentials analysis). The final sample included 12 women and 10 men aged between 18 and 29 years (mean age = 23.39; SD = 3.07). Participants who took part in the experiment were those who were not rejected after having been screened for the following exclusion criteria: Being left-handed, a medical condition requiring daily medication, a previous history of alcohol and/or substance abuse, psychiatric illness, a diagnosed learning disability, neurological history (seizure, central nervous system neoplasm or brain tumour) or a traumatic brain injury. As the SRT task involves learning of a repeating motor sequence of finger movement, participants were also screened for rheumatic diseases. All participants were right handed and had a normal vision, before or after correction. The study was approved by the local ethics committee of the Université du Québec à Trois Rivières and all participants provided written informed consent prior to testing. Participants received a financial compensation of *$*30 CDN for their participation.

### Procedure

The experimental protocol included the administration of a general health questionnaire and a SRT task during which continuous EEG activity was recorded from 64 scalp electrodes. Participants came to the laboratory for a single 90-minute testing session. The health questionnaire was administered to obtain demographic as well as medical history information.

#### Serial reaction time task

**Figure 6:**
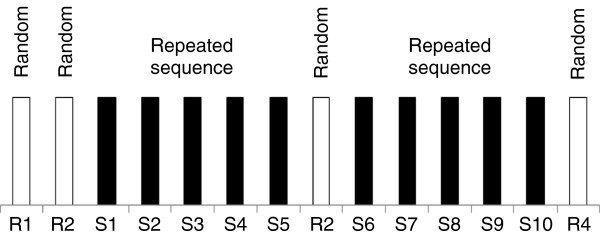
**Graphical representation of the blocks of stimuli included in the SRT task.** Blocks designated R contained a new random sequence whereas those designated S contained the same repeated sequence (see text). Learning of the repeated sequence was assessed by comparing mean response time (RT) across the last two blocks (S10 vs. R4).

Participants were seated on a straight back chair with elbows flexed at an angle of 90°. They performed a SRT task [27] running under E-Prime 2.0 (Psychology Software Tools Inc., Sharpsburg, PA, USA). A GO signal was displayed on the computer screen and consisted of one cross and 3 dashes evenly spaced horizontally and centered on a fixation point, all appearing simultaneously. The cross and dashes were 0.34° wide and positioned at a visual angle of 5.5° and 2° to the left and at 5.5° and 2° to the right of the fixation point. The fixation point always remained present while the position of the cross varied across trials among the 4 possible locations and indicated the required key press. Participants were instructed to respond as fast as possible to the position of the cross by pressing the corresponding key with the predetermined finger of the right hand (index finger for key 1, middle finger for key 2, ring finger for key 3, and little finger for key 4). A correct key press was required for the next trial to appear on the computer screen. RT was defined as the time interval between stimulus onset and the corresponding key press. Participants performed a total of 14 blocks separated by pauses, using the sequence structure shown in Figure 6. Ten of these 14 blocks included a repeated sequence that consisted of 10 presentations of the same 12-item sequence for a total of 120 key presses per block. Participants were instructed to perform the task only with their dominant right hand and to keep the appropriate finger on each predetermined key at all times. The two initial blocks, the eighth block and the last block consisted of stimuli presented in a random order (random blocks) that differed from the predetermined repeating sequence. The first two random blocks (R1 and R2) were provided for participants to get familiar with the task. Blocks 3 to 7 and 9 to 13 corresponded to training blocks during which participants were presented with the following predetermined, repeating 12-item sequence (sequence S: 4--2--3--1--1--3--2--1--3--4--2--4). Blocks were named according to their respective order preceded by the letter “S” for those including the repeated sequence (S1 to S10) and by the letter “R” for those presenting the stimuli in a random order (R1 to R4). Sequence-specific learning was computed as the difference in mean RT between the last sequence block (S10) and the last random block (R4) [45], as shown in Figure 6.

#### Electrophysiological data acquisition

The EEG was recorded from 64 active Ag/AgCl electrodes (ActiCAP, Brain Products) positioned according to the standard 10–10 system, with the exception that TP9 and TP10 electrode sites were not used, and replaced by electrodes placed at the mastoids. All electrodes were recorded with a left-mastoid reference, and the data were re-referenced offline to the algebraic average of the left and right mastoids [46]. Additional cutaneous electrodes were used to monitor electrooculographic activity; two placed on external canthi to record the horizontal electrooculogram (HEOG) and two placed on infra/supraorbital regions to record the vertical electrooculogram (VEOG). All electrode impedances were kept below 15 kΩ. The EEG was digitized at 500 Hz, high-pass filtered at 0.01 Hz and low-pass filtered at 225 Hz during the recording.

#### Event-related potentials analysis

Using the software Brain Vision Analyser 2.0 (Brain Products, Germany), signals were further high-pass filtered at 0.1 Hz and low-passed at 20 Hz offline. Consistent with the literature, the ERN waveform was analyzed at pooled electrodes Cz and FCz, which are in the vicinity of the peak of the scalp distribution of the ERN component, and hence typically provides an optimal site for statistical analysis in response to error commission [4, 5, 12, 32]. Trials with eye blinks (VEOG > 100 μV), large horizontal eye movements (HEOG > 35 μV), and/or artefacts (>80 μV) at one of the 62 recording electrodes (64 electrodes with the exception of two reference electrodes) were excluded from further analysis using an automated screening procedure. The EEG was segmented relative to the onset of each response in the modified SRT task to create response-locked epochs of 600 milliseconds (ms) that included a 300 ms pre-response period. Epochs were baseline corrected relative to the mean signal amplitude between -200 and 0 ms prior to the response. Separate averaged waveforms were computed for error trials (ERN) and correct trials (correct-related negativity or CRN). ERN components were obtained by subtracting neurophysiological brain activity for correct trials from that elicited by error trials (i.e., ERN-CRN) [24]. For each subject, three ERN difference waveforms were computed: 1- averaged ERN recorded during the first four learning blocks of the modified SRT task (corresponding to blocks S1 to S4); 2- averaged ERN recorded from the last four learning blocks of the task (corresponding to blocks S7 to S10) & 3- averaged ERN recorded from all learning blocks of the task (S1 to S10). Importantly, research shows that the ERN waveform component is reliable with as few as six error trials [47, 48]. Peak amplitude of these three averaged ERN-CRN difference waveforms was determined using a semiautomatic mode as the most negative sample point recorded within a predefined time window of 0-100 ms after the response. Mean ERN amplitude (μV) was then determined as the mean value around the peak, including 12 sample points before and after the peak (for a total of 25 points), corresponding to a time window of 50 ms. The latency of ERN components corresponded to the time point at which the ERN was of maximal amplitude. ERN amplitude change during SRT task performance (as well as ERN latency change) was quantified as the difference between the averaged ERN recorded from the first four learning blocks and the averaged ERN recorded from the last four learning blocks (S_1-4_ – S_7-10_).

#### Statistical analyses

Paired *t* tests were used to compare performance scores, ERN amplitude and ERN latency recorded in the S_1-4_ learning blocks and S_7-10_ learning blocks. Two-tailed Pearson correlations were drawn in order to test potential associations between ERN amplitude change and SRT task performance improvement. *P* < 0.05 was considered to be statistically significant. All statistical analyses were conducted using IBM SPSS Statistics for Windows, Version 21.0 (IBM, United States).

## Abbreviations

ACC: Anterior cingulate cortex
BDNF: Brain-derived neurotrophic factor
CRN: Correct-related negativity
EEG: Electroencephalogram
ERN: Error-related negativity
ERP: Event-related potential
HEOG: Horizontal electrooculogram
M1: Primary motor cortex
OCD: Obsessive-compulsive disorder
RT: Response time
SMA: Supplementary motor area
SRT: Serial reaction time
TBI: Traumatic brain injury
VEOG: Vertical electrooculogram.
